# Supplementary material for: HIVprotI: an integrated web based platform for prediction and design of HIV proteins inhibitors
Source: J Cheminform. 2018 Mar 9;10:12. doi: 10.1186/s13321-018-0266-y (PMC5845081; doi:10.1186/s13321-018-0266-y)
Supplement: Supplementary file 2 — Additional file 2. Source code of HIVProtI web server. [file 13321_2018_266_MOESM2_ESM.zip › HIVprotI_Source-code/hivprotI/weka-3-6-6/doc/weka/classifiers/meta/package-frame.html]

weka.classifiers.meta


weka.classifiers.meta

Classes 
  
AdaBoostM1
  
AdditiveRegression
  
AttributeSelectedClassifier
  
Bagging
  
ClassificationViaClustering
  
ClassificationViaRegression
  
CostSensitiveClassifier
  
CVParameterSelection
  
Dagging
  
Decorate
  
END
  
FilteredClassifier
  
Grading
  
GridSearch
  
LogitBoost
  
MetaCost
  
MultiBoostAB
  
MultiClassClassifier
  
MultiScheme
  
OrdinalClassClassifier
  
RacedIncrementalLogitBoost
  
RandomCommittee
  
RandomSubSpace
  
RegressionByDiscretization
  
RotationForest
  
Stacking
  
StackingC
  
ThresholdSelector
  
Vote
